# One Conservation: the integrated view of biodiversity conservation

**DOI:** 10.1590/1984-3143-AR2021-0024

**Published:** 2021-06-02

**Authors:** Cristiane Schilbach Pizzutto, Helen Colbachini, Pedro Nacib Jorge-Neto

**Affiliations:** 1 Faculdade de Medicina Veterinária e Zootecnia, Universidade de São Paulo, São Paulo, SP, Brasil; 2 Instituto Reprocon, Campo Grande, MS, Brasil; 3 Aquário de São Paulo, São Paulo, SP, Brasil

**Keywords:** in situ, ex situ, sustainability, wildlife, reproduction

## Abstract

The current global situation requires urgent decision-making to reverse processes of mass extinction of thousands of species. As a way of showing the importance of joint actions in this process, we aim to present the concept of One Conservation as a new proposal for the integration of sustainability, *in situ* and *ex situ* conservation for the restoration of ecosystems. According to the United Nations, we are beginning the decade of ecosystem restoration and in association with the International Union for Conservation of Nature guidelines, we can join efforts in the conservation of the planet. The survival of many species of wild animals depends on the management of populations currently maintained in *ex situ* conditions (under human care). To facilitate the exchange of genetic material between *in situ* and *ex situ* populations, reproductive biotechniques have become a great tool, making it possible to restore species in their natural environments. For effective conservation to occur, there must be an integrated view of the problem as a whole, and action for solutions must take place jointly by different spheres of society. Even more, conservation must be carried out by the public sector, the private sector, the third sector, and not less importantly, the agricultural sector. Therefore, One Conservation is defined as an interconnection between *ex situ* and *in situ* conservation plans, anthropic actions in the environment, and research in different areas that encompass conservation.

## Introduction

The human species, despite considered very young compared to other animal species (Gershwin, 2016), is endowed with complex intellectual characteristics and abilities (Gabora and Russon, 2011). Even so, humankind did not know how to work harmoniously with the man-animal-environment relationship. Unlike protectionism – which focuses on the integral protection of nature, without human interference –, conservationism contemplates saving the environment through sustainable and harmonious use of nature by humankind (Padua, 2006).

We are experiencing the sixth mass extinction of wildlife on Earth and human actions – such as deforestation, wildfire, mining, urbanization, habitat fragmentation, etc. – are rapidly changing the conservation status of several animal and plant species (Ceballos et al., 2015). Associated with all these problems, stochastic events have contributed to making animal populations smaller and isolated, leading them to fast and no return disappearing (vortex of extinction; Keller, 2002). Conservation actions are carried out around the world, reaching varying degrees of effectiveness. However, the lack of an integrated view between actions often limits their effectiveness.

**Figure 1:**
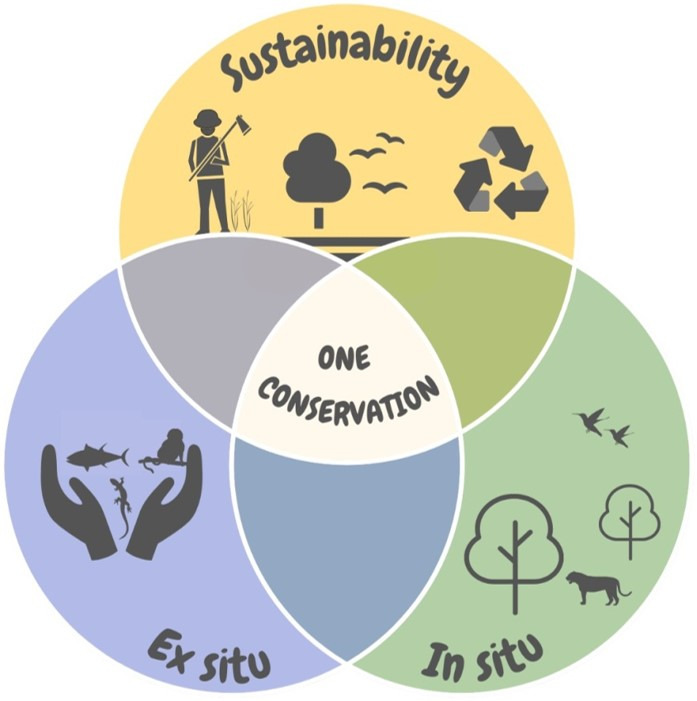
Venn Diagram – One Conservation is at the intersection of sustainability, *ex situ*, and *in situ* conservation. Illustrator: Carolina Schilbach Pizzutto

Due to an urgent need to accelerate global actions focused on restoring degraded ecosystems (Waltham et al., 2020), the United Nations Decade on Ecosystem Restoration (2021 to 2030) was instituted to prevent, halt and reverse the degradation of ecosystems worldwide. It is a decisive moment for taking crucial decisions and establishing a partnership with all the stakeholders for the creation of conservation action strategies. Looking at this scenario, we aim to present the concept of One Conservation as a new proposal for the integration of sustainability, *in situ* and *ex situ* conservation for the restoration of ecosystems (Figure 1).

## Contextualizing the concept

The perception of the integration between human and animal health began in the 19th century with Rudolf Virchow. It was achieved through the definition of the One Health concept as a worldwide strategy for the integration of human, animal, and environmental health through communication and collaboration between different professionals related to this area (Pinillos et al., 2015). Developing this concept, Pinillos et al. (2015) suggest a more complex approach, defining the One Welfare concept by recognizing the interconnections between animal welfare, human welfare, and environmental conservation.

Recognizing the urgency of creating a single global strategy to reverse social inequality and environmental degradation, the United Nations defined seventeen Sustainable Development Goals for its 2030 agenda (UN General Assembly, 2015). It also defined 2021-2030 as the Decade of Ecosystem Restoration.

The COVID-19 pandemic further highlighted the interrelationship between the health and well-being of man, animals, and the environment. And it is in this context that we suggest the creation of an even more comprehensive concept: The One Conservation concept, which encompasses One Health and One Welfare concepts and recognizes the interdependence of human species, animal species and conservation ecosystems.

Sustainability means guaranteeing the rights and well-being of humans without exhausting or diminishing the capacity of Earth's ecosystems to sustain life, or at the expense of the well-being of others (FAO, 2018). It is a multidimensional concept that encompasses the integrity of the environment, the well-being of society, economic resilience, and good governance. The integration of actions that facilitate the restoration of ecosystems is an emergency.

## *In Situ* + *Ex Situ*

Restoring populations is not an easy task and integrated actions between *in situ* and *ex situ* conservation are essential. Multidisciplinary joint actions, with public and private partnerships and from different segments of society must be performed. *In situ* conservation consists of strategies for the conservation of ecosystems and natural habitats and the maintenance and recovery of viable species by natural means. *Ex situ* conservation – under human care – focuses on the preservation and recovery of species through captive populations.

The *ex situ* maintenance of wild fauna is a target of criticism in several countries around the world, but currently this collection of animals under human care may be the only alternative for the survival of many species. The existence of a zoo or an aquarium is only justified by its ability to play its role within the four pillars established by Hediger (1950): conservation, research, education, and entertainment. Likewise, *in situ* conservation depends primarily on understanding and eliminating the factors of decline in wild populations. Thus, *in situ* conservation is not efficient when the species is below the minimum viable population. The synergism of *ex situ* and *in situ* actions reinforces not only the conservation pillar but also the need for the existence of these institutions.

Conservation, whether *in situ* or *ex situ*, involves maintaining minimum viable populations. A minimum viable population is the smallest population size with a high probability - 90% or more - of persisting for the next 100 years (Shaffer, 1990, 1981). Reed et al. (2003) estimated that the average minimum viable adult population size for most free-living vertebrate species is 7,000 individuals. In captivity, this number varies between 500 and 1,000 animals (Frankham et al., 2014; García-Dorado, 2015). Therefore, the IUCN guidelines (IUCN/SSC, 2014) include an insurance population among the strategies for an *ex situ* conservation program.

Often the only alternative for the survival of endangered species is the adoption of integrated *ex situ* conservation strategies for zoos, aquaria, and scientific breeding centers. At the same time, fundamental research in understanding the diversity of characteristics not only of these species but of practically the entire animal kingdom occurs in these institutions. For Pizzutto (2020), many of these characteristics come from the mechanisms involved in the regulation of the various physiological and behavioral models that have evolved from different adaptation strategies, as a result of selective pressures exerted by the modified environment or not.

When we manage wild animals intending to provide positive experiences for them within the domains of health, nutrition, environment, and behavioral interactions, we seek more than an adaptation to their environment, but that they thrive as a species and reach their level of ideal well-being, within the fifth domain – the mental. For Mellor et al. (2020), the effects in the fifth domain are generated by the brain processing of external stimuli from sensory inputs that allow animals to interact with their environments, achieving or not achieving their selected objectives. Behavioral interactions state the positive and/or negative emotions experienced by animals and will dictate the condition of well-being.

Everything reinforces how much conservation depends on well-being. Ensuring physical, mental, and emotional health to the animals are ways to guarantee a good aptitude for a species so that it can thrive, becoming able and viable in population management programs. A species kept *ex situ* fit and viable is a big step towards partnering with *in situ*. If these populations kept under human care can carry out the genetic exchange with free-living species and vice versa, zoos, aquaria, and scientific breeding centers will be exercising their role as a metapopulation and enabling an increase in genetic variability.

The view that *in situ* conservation actions should be prioritized before *ex situ* is outdated and obsolete. Both are equally important, and the One Conservation concept proposes that they should be conducted in an integrated way. The argument that *ex situ* conservation is expensive is limited way, as it disregards that private corporations, through zoos, aquaria, and scientific breeding centers, are extremely important players in the conservation milieu and invest and pay for *ex situ* actions. Equally, *in situ* conservation needs to be conducted realistically, based on facts. Conducting it only with optimism can result in the extinction of species or populations in certain areas or biomes by not sticking to the facts and procrastinating effective actions. Therefore, waiting passively for further studies may harm even more species in a critical conservation situation.

## Reproductive biotechnology for One Conservation

The genetic exchange between *in situ* and *ex situ* populations can be facilitated by reproductive biotechnologies, such as artificial insemination, *in vitro* fertilization, embryo transfer, and even cloning and transgenics. According to Baldassarre et al. (2015), developing these biotechnologies would be critical for allowing their use as a tool for rebuilding balance in animal numbers for such endangered species.

The development of reproductive biotechnologies for wildlife species encompasses three main thematic: basic knowledge of the species (e.g., reproductive physiology, reproductive and copulatory behavior); male biotechniques (e.g., efficient semen collection and cryopreservation); and female biotechnologies (e.g., insemination techniques, ovum pick-up and *in vitro* embryo production).

Germplasm banks (biobanking) are being created all over the world to store biological material of various species maintained both *in situ* and *ex situ*. This can ensure the preservation of rare and endangered species in the future (Comizzoli, 2017). Associated with the studbook information and effective population management programs mentioned above, biobanking is a great ally of conservation. Since reproduction can be performed without the need to transport the animal, biobanking can safeguard species and allows directed breeding (decreasing the spread of diseases and injuries, eliminating genetic incompatibilities).

A great example is the Frozen Zoo, located at the Beckman Center for Conservation Research of the San Diego Zoo Wildlife Alliance. It is currently the largest and most diverse collection of germplasm in the world. It has the potential to produce offspring using reproductive biotechnologies and perhaps to rescue species like the northern white rhinoceros from the brink of extinction.

Strategies for the conservation of Brazilian fauna thru germplasm cryopreservation and storage in biobanks are being taken (Machado et al., 2016; Miranda et al., 2019; Praxedes et al., 2018a). Gametes of different species are being collected and cryopreserved (Araujo et al., 2020; Carelli et al., 2017; Silva et al., 2020b; Silva et al., 2019a, b) as well as somatic cells (Praxedes et al., 2018b) and fibroblasts and different tissues such as testicular (Silva et al., 2020a), ovarian (Campos et al., 2019b), skin from post-mortem animal (Machado et al., 2017; Santos et al., 2021), preantral follicles (Campos et al., 2019a) and even somatic feather follicle cell (Cardoso et al., 2020). Although these biotechnologies are extremely promising for the conservation of species, it is important to note that many of them have not yet been successful in free-living animals.

## One Conservation: the integrated view

For effective conservation to occur, there must be an integrated view of the problem, and action for solutions must take place jointly by different spheres of society. Even more, conservation must be carried out by the public sector, the private sector, the third sector, and not less importantly, the agricultural sector. Society must be actively involved in conservation, from the housewife to the president of the country. Some of the professionals that work directly with conservation are animal scientists, biologists, botanists, conservation managers and planners, ecotourism guides, environmental education specialists, freshwater fishery biologists, landscape ecologists, marine biologists, park rangers, veterinarian, wildlife filmmaker, and photographer – the latter two being the great spokespersons for information and the state of nature conservation for society.

The insertion of farmers and ranchers in environmental care is essential for One Conservation, given the size of land owned by this category. Despite the intentions of the agribusiness sector, the support of conservation technicians is essential for taking positive action in sustainable and efficient rural production. Therefore, conservationists need to see agribusiness as an opportunity for sustainable conservation, not as a villain, and work together targeting One Conservation.

Despite the importance of all these actions being already known, the difficulty of integrating them in favor of conservation is still a major problem for the effectiveness of the different management plans. One solution would be to compile all information obtained from fauna projects on a single digital platform of technical and scientific knowledge and open access to scientific and civil society.

Another key point would be the maintenance of updated records of animals – through Zoological Information Management System, Studbooks, or other tools – maintained by institutions associated with Zoos and Aquaria Associations. These associations should also act as contact facilitators between different zoos and aquaria, as well as scientific breeding centers, research, and/or also *in situ* conservation institutions that know reproductive biotechnologies.

Registering and making these animals available to collaborate with One Conservation Programs should be mandatory and conditional for the certification process of zoos and their maintenance as associate members. These certifications would be an important step to ensure that populations kept in ex situ conditions are under the management of institutions that meet recognized protocols for animal care, and that these institutions are demonstrably committed to the conservation, increasing the trust of *in situ* research groups and working seamlessly with zoos and aquariums.

## Conclusions

Synergism between different lines of research is essential for the conservation of any species and nothing is effective in isolation. In this way, different groups (e.g., behavior, *in situ* monitoring, environmental education, conflict resolution, reproduction, genetics, etc.) must work together for the same purpose: conservation. Conservation is essential to include environmental education actions for the coexistence and resolution of conflicts.

One Conservation is defined as an interconnection between *ex situ* and *in situ* conservation plans, anthropic actions on the environment (sustainability), and research in different areas that encompass conservation.
